# Apolipoprotein E isoform does not influence trans-synaptic spread of tau pathology in a mouse model

**DOI:** 10.1177/23982128231191046

**Published:** 2023-08-17

**Authors:** Caitlin Davies, Jane Tulloch, Ellie Yip, Lydia Currie, Marti Colom-Cadena, Susanne Wegmann, Bradley T Hyman, Lewis Wilkins, Monique Hooley, Makis Tzioras, Tara L Spires-Jones

**Affiliations:** 1 Centre for Discovery Brain Sciences and UK Dementia Research Institute, The University of Edinburgh, Edinburgh, UK; 2 German Center for Neurodegenerative Diseases (DZNE), Berlin, Germany; 3 Department of Neurology, Massachusetts General Hospital, Charlestown, MA, USA; 4 Harvard Medical School, Boston, MA, USA

**Keywords:** Alzheimer, tauopathy, tau, synapse, apolipoprotein E, array tomography

## Abstract

A key hallmark of Alzheimer’s disease (AD) is the accumulation of hyperphosphorylated tau in neurofibrillary tangles. This occurs alongside neuroinflammation and neurodegeneration. Pathological tau propagates through the AD brain in a defined manner, which correlates with neuron and synapse loss and cognitive decline. One proposed mechanism of tau spread is through synaptically connected brain structures. Apolipoprotein E4 (*APOE4*) genotype is the strongest genetic risk factor for late-onset AD and is associated with increased tau burden. Whether the apolipoprotein E (*APOE*) genotype influences neurodegeneration via tau spread is currently unknown. Here, we demonstrate that virally expressed human tau (with the P301L mutation) injected into mouse entorhinal cortex at 5–6 months or 15–16 months of age spreads trans-synaptically to the hippocampus by 14 weeks post-injection. Injections of tau in mice expressing human *APOE2*, *APOE3* or *APOE4*, as well as *APOE* knock-outs, showed that tau can spread trans-synaptically in all genotypes and that *APOE* genotype and age do not affect the spread of tau. These data suggest that *APOE* genotype is not directly linked to synaptic spread of tau in our model, but other mechanisms involving non-cell autonomous manners of tau spread are still possible.

## Introduction

Accumulation of tau aggregates in neurofibrillary tangles is one of the hallmark pathologies of Alzheimer’s disease (AD) and correlates strongly with disease progression, neurodegeneration and cognitive decline (Arriagada et al., 1992; Bejanin et al., 2017; Giannakopoulos et al., 2003; Gordon et al., 2018; Honer, 2003; La Joie et al., 2020; Nelson et al., 2012). Progressive accumulation of tau is highly stereotypical, is used to stage AD (Braak and Braak, 1991) and has led to the hypothesis that pathological tau propagates from one brain region to another along axonal projections. Indeed, internalised tau aggregates can be transported along axons and dendrites (Ahmed et al., 2014; Takeda et al., 2015; Wu et al., 2013) and could leave one neuron and be internalised by another (Calafate et al., 2015), indicating pathological tau may spread neuron-to-neuron.

Injections of both synthetic tau fibrils and tau-containing brain extract into the mouse brain can induce the formation of tau aggregates that propagate to connected areas and recapitulate the hallmark lesions of the associated tauopathy (Ahmed et al., 2014; Boluda et al., 2015; Clavaguera et al., 2009, 2013; Guo et al., 2016; Iba et al., 2013, 2015; Kaufman et al., 2016; Narasimhan et al., 2017; Peeraer et al., 2015; Sanders et al., 2014; Stancu et al., 2015). Moreover, when human tau is selectively expressed in the entorhinal cortex (EC), using transgenic or virally mediated models, human tau propagates to synaptically connected dentate gyrus (DG) neurons (De Calignon et al., 2012; Harris et al., 2012; Liu et al., 2012; Pickett et al., 2017; Wegmann et al., 2015, 2019), suggesting tau traverses monosynaptic circuits in living brain. Importantly, in vivo, human tau was seen to propagate through intact synapses without overt degeneration (Pickett et al., 2017), while in vitro, synaptic contacts between neurons facilitated propagation of pathological tau (Calafate et al., 2015).

Emerging evidence from post-mortem brains (DeVos et al., 2018) and positron emission tomography (PET) scans (Franzmeier et al., 2020; Hoenig et al., 2018; Jones et al., 2017; Vogel et al., 2020) from individuals with AD have begun to hint that tau spreads through functionally connected neural networks in human brains; however, most of that data are correlative. Tau pathology accumulates in EC during ageing even in cognitively normal elderly adults, but in AD, spread beyond EC is thought to be important in driving neurodegeneration and cognitive decline (Adams et al., 2019; Hyman et al., 1984).

In humans, Apolipoprotein E (*APOE*) exists as three major allelic variants (*APOE2*, *APOE3* and *APOE4*), and it is the major genetic determinant of susceptibility to development of late-onset AD (Coon et al., 2007; Corder et al., 1994; Farrer et al., 1997; Saunders et al., 1993). Compared to the most frequent allele, *APOE3*, inheritance of *APOE4* increases risk of developing AD and reduces the age of onset in a gene dose-dependent manner (Corder et al., 1994; Farrer et al., 1997). In both mice and humans, *APOE4* is associated with increased tau phosphorylation (Brecht et al., 2004; Tesseur et al., 2000), pathological tau burdens (Altmann et al., 2014; Kumar et al., 2015; Sabbagh et al., 2013; Tiraboschi et al., 2004), neurodegeneration (Litvinchuk et al., 2021; Shi et al., 2017) and gliosis (Minett et al., 2016; Overmyer et al., 1999); however, not much is known regarding its effects on the spread of tau (Tzioras et al., 2019).

Overall, there is a lack of consensus regarding the effect of *APOE* isoform on tauopathy in model systems and in human AD brain, although there are indications that *APOE4* may preferentially influence regions involved in the initial spread of tau. In this study, we tested the hypothesis that *APOE* isoforms differentially regulate trans-synaptic tau spread. To do this, using mice with genetic ablation of murine *Apoe* or knock-in of human *APOE2*, *3* or *4* isoforms into the mouse *Apoe* locus, we examined trans-synaptic spread of human tau virally expressed in EC, which is a brain region affected early by tau pathology in AD.

## Methods

### Mice

Male and female *APOE2*, *APOE3* and *APOE4* targeted replacement (TR) and *Apoe* knock-out (KO) mice were acquired from Taconic (Germantown, NY, USA; model no.: #1547, #1548, #1549 and #APOE, respectively) and bred in-house in homozygous colonies. Mice were intermittently genotyped to ensure no errors in breeding. *APOE2*-TR, *APOE3*-TR and *APOE4*-TR mice express the respective human *APOE* isoform under the control of the murine *Apoe* regulatory sequences (Knouff et al., 1999; Sullivan et al., 1997). In *Apoe* KO mice, the endogenous mouse *Apoe* gene is inactivated by insertion of a neomycin cassette (Piedrahita et al., 1992). All mice were maintained on a C57BL/6 background. Male and female adult C57/BL6 mice, bred in-house, were used for assessment of stereotaxic injection co-ordinates and adeno-associated virus (AAV) concentration. Animals were housed in a 12:12-h light:dark cycle and had ad libitum access to food and water. Prior to surgical procedures, animals were group-housed and, where possible, were returned to original group-housing post-surgery. Animals of both sexes were used, and sex was considered as a biological variable in statistical models.

Animal experiments were performed in accordance with national and institutional guidelines including the Animals (Scientific Procedures) Act 1986, the Council Directive 2010/63EU of the European Parliament and the Council of 22 September 2010 on the protection of animals used for scientific purposes. All experiments had full UK Home Office ethical approval. Experimenters were blind to mouse genotype during immunohistochemistry, image acquisition and image analysis.

### Adeno-associated virus stereotaxic injection

**Figure 1.**
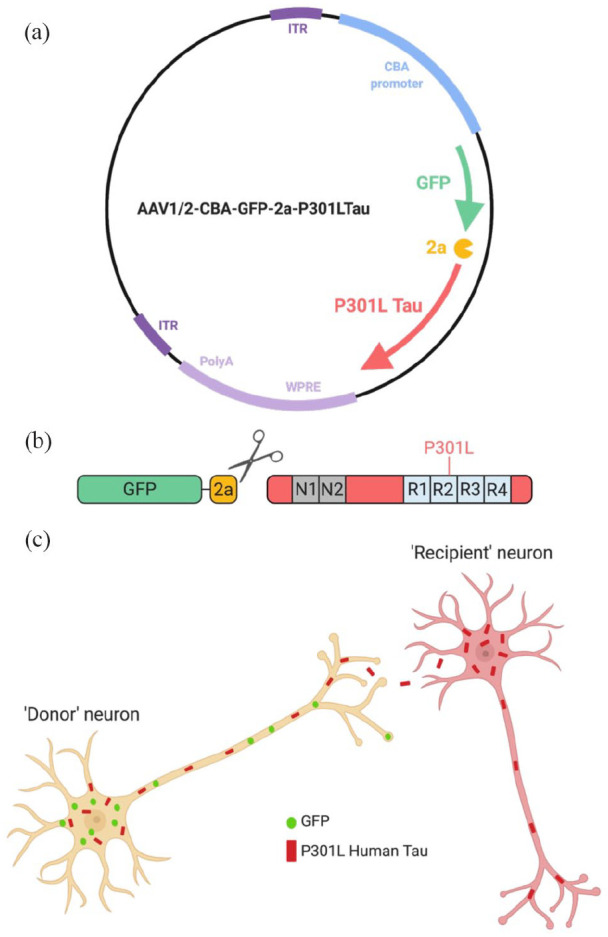
Tau adeno-associated virus (AAV) used for studying tau propagation in the mouse brain. The AAV vector (a) encodes green fluorescent protein (GFP), a translation interrupting 2a peptide and full-length mutant (P301L) human tau in one RNA transcript. Due to the ‘self-cleaving’ 2a peptide, GFP-2a and full-length P301L tau are expressed as individual proteins (b). Immunostaining for GFP and human tau allow for discrimination between ‘donor neurons’ transduced with the AAV and ‘recipient neurons’ that have received P301L tau through spreading mechanisms (c). Source: Image created using BioRender.

The AAV used in this study was produced by the viral vector core at the University of Edinburgh and was modelled on an AAV vector acquired from Dr Suzanne Wegmann et al. (2017). The AAV used encodes, in one RNA transcript, green fluorescent protein (GFP), a translation interrupting 2a peptide and full-length mutant (P301L) human tau. Expression of the construct is driven by the ubiquitous chicken beta-actin (CBA) promoter and is enhanced by a woodchuck post-translational regulatory element (WPRE) and polyA sequence (AAV1/2-CBA-GFP-2A-P301Ltau-WPRE). Due to the ‘self-cleaving’ properties of the 2a peptide (Szymczak et al., 2004), upon translation GFP-2a and P301L tau are expressed as individual proteins in an equimolar ratio in infected cells. This allows for the investigation of tau propagation in the mouse brain by immunohistochemical detection of ‘donor neurons’ transduced with the AAV and non-transduced ‘recipient neurons’ that have received P301L tau through spreading mechanisms. When staining for GFP and human tau, donor neurons can be identified by immunolabelling of both proteins, while recipient neurons are immuno-positive for human tau but negative for GFP (Figure 1).

GFP-2a-P301Ltau AAV was bilaterally injected into the superficial layers of the entorhinal cortices of 5- to 6-month-old (adult) and 15- to 16-month-old (aged) *APOE2/3/4*-TR and *APOE*-KO mice. All surgical procedures were conducted under aseptic conditions. Mice were anaesthetised using isoflurane (3%–5% induction; 1%–2% maintenance) and placed into a stereotaxic frame. Body temperature was maintained at 37°C throughout using a homeothermic blanket (Harvard Apparatus, Holliston, MA, USA). Following removal of the fur, a midline incision of the skin was made and burr holes were drilled through the skull at the injection sites. Co-ordinates for injection sites were: anterior/posterior = −4.70 mm; medial/lateral = ±4.25 mm; dorsal/ventral = −2.50 mm (from the brain surface); using a 20° (in the lateral direction, away from the midline). Using a pulled micropipette, 1 μL of AAV (concentration = 5 × 10^8^ vg/μL) was injected into the EC of each hemisphere over a period of at least 5 min (0.2 μL/min). Upon completion of the injection, the micropipette was left in place for 5 min to enable diffusion of the AAV solution before being slowly removed. The skin incision was sutured, and mice recovered in a clean home cage placed inside a recovery chamber maintained at 37°C. Prior to surgery, mice were habituated to oral administration of analgesia in the form of cubes of jelly. During surgery, mice received subcutaneous injection of 1 mL sterile saline and the analgesic buprenorphine (0.05 mg/kg) and received continuous analgesia in the form of cubes of jelly for at least 3 days post-injection.

To determine accurate stereotaxic co-ordinates consistently targeting superficial layers of the EC in our mouse colonies, a blue dye (Toluidine Blue) and a fluorescent dye (Texas Red) were injected into the EC of WT mice of the same background strain. Brains were sectioned in the sagittal plane and imaged to establish co-ordinate accuracy. Tested co-ordinates were identified from the literature and modified based on observed accuracy or inaccuracy. After multiple rounds of modification, the co-ordinates: A/P = −4.7 mm, M/L = ±4.25 mm, D/V = −2.50 mm; with the micropipette tilted 20° in the M/L direction away from the midline, consistently and accurately targeted LII/III of the EC To ensure that any differences between WT C57/BL6 and APOE-KI and -KO mice did not influence injection accuracy, co-ordinates were further confirmed in APOE-KI and -KO mice (Supplementary Figure 1). Co-ordinate accuracy was not affected by sex.

To confirm viral expression of GFP and human tau and establish an appropriate tau (P301L)-AAV concentration for stereotaxic injection, three different AAV concentrations (1 × 10^8^, 5 × 10^8^ and 1 × 10^9^ vg/μL) were injected bilaterally into the somatosensory cortices of WT C57/BL6 mice. Mice were culled after 3 weeks, to allow time for viral expression, and brains sectioned in the coronal plane. Sections were stained for GFP and human tau (Tau13). Staining for both GFP and Tau13 was observed (Supplementary Figure 1), confirming expression of the viral construct. Although not quantified, by eye, it was clear that injection with viral concentration of 1 × 10^8^ vg/µL resulted in minimal transduction of cells around the injection site. Concentrations of 5 × 10^8^ vg/μL and 1 × 10^9^ vg/µL resulted in a greater and sufficient number of transduced cells (Supplementary Figure 1). Considering expression of GFP can be cytotoxic (Ansari et al., 2016) and as there was no obvious difference in transduction between the two higher concentrations, the lower concentration of 5 × 10^8^ vg/μL was selected for use.

We chose an incubation time of 14 weeks to allow viral expression and maximise the chances of detecting spread of tau pathology based on our previous studies using the same virus which shows tau spreading at 6, 10 and 12 weeks which is variable depending on mouse genetic background strain (Dujardin et al., 2022; Wegmann et al., 2019). Fourteen weeks post-injection, mice were deeply anaesthetised with isoflurane (5%) and a lethal dose of Euthatal (i.p.), before transcardial perfusion with cold 10 mM phosphate-buffered saline (PBS). The brain was removed and hemisected. One hemisphere was randomly selected and placed into fixative containing 4% paraformaldehyde and 15% glycerol in 10 mM PBS for use in free-floating immunohistochemistry. Hemispheres were left to fix and cryoprotect for at least 5 days. The other hemisphere was submerged in array tomography fixative containing 4% paraformaldehyde and 2.5% sucrose in 10 mM PBS for 2–3 h for use in array tomography experiments.

### Immunohistochemistry and imaging of tau spread in free-floating sections

Cryoprotected hemispheres were mounted onto a Leica SM2010 R sliding microtome (Leica, Wetzlar, Germany) using water and optimal cutting temperature (OCT) compound (Agar Scientific, Stansted, UK) and allowed to freeze using dry ice. Once frozen, 50 μm horizontal sections were cut and sections containing EC and hippocampus were collected such that each Eppendorf contained every 10th section. Sections were stored in 15% glycerol at −20°C until they were needed. A tube containing every 10th section was randomly selected and thawed. Sections were washed 3× in TBS and permeabilised in 0.2% Triton-X (TX) in TBS for 20 min at room temperature. Sections from young mice were then blocked in 5% normal goat serum (NGS) in 0.2% TX in TBS for 1 h at room temperature. Primary antibodies (anti-GFP Abcam13970, 1:1000 and anti-human tau Tau13 Biolegend 835201 1:1000) diluted in 3% NGS in 0.2% TX in TBS were applied and incubated overnight at 4°C. Sections were washed thoroughly in TBS, and secondary antibodies (goat anti-chicken IgY-AlexaFluor488, Abcam ab150169l goat anti-mouse IgG1-AlexaFluor647, Invitrogen A-21240), diluted in 3% NGS, were applied for 1.5 h at room temperature. Sections were counterstained with 0.01 mg/mL 4’,6-diamidino-2-phenylindole (DAPI) to visualise nuclei. To quench background autofluorescence, sections were treated with Autofluorescence Eliminator (EMD Millipore Corp., Billerica, MA, USA) according to the manufacturer’s protocol for 5 min. Sections were then mounted onto glass slides (VWR, Radnor, PA, USA) and coverslipped using Immu-mount™ mounting media (Thermo Fisher Scientific, Waltham, MA, USA). Following permeabilisation, to obtain improved staining, sections from old mice underwent antigen retrieval by pressure cooking for 2 min in 10 mM citric acid in ddH_2_0 (pH 6.0). Slices were washed in TBS and blocked in 5% milk in 0.2% TX in TBS for 1 h at room temperature. Primary antibodies (anti-GFP Abcam13970, 1:1000 and anti-human tau Tau13 Biolegend 835201 1:1000) diluted in 3% milk in 0.2% TX in TBS were applied and incubated overnight at 4°C. Sections were washed thoroughly in TBS and secondary antibodies (goat anti-chicken IgY-AlexaFluor488, Abcam ab150169l goat anti-mouse IgG1-AlexaFluor647, Invitrogen A-21240), diluted in 3% milk, were applied for 1.5 h at room temperature. Sections were counterstained with 0.01 mg/mL DAPI to visualise nuclei. Sections were then mounted on glass slides (VWR) and coverslipped using Immu-mount™ mounting media (Thermo Fisher Scientific).

Stained sections were visualised using a ZEISS Axio Imager Z2 stereology microscope (Carl Zeiss, Oberkochen, Germany). Tilescan images of each individual section were obtained using a 10× objective and MBF Biosciences Stereo Investigator software (MicroBrightField, Inc., Williston, VT, USA). Exposure times for each channel were kept consistent between mice, and the plane of focus was the same between channels. Images were converted to tiff format and each channel was segmented using custom MATLAB scripts. Segmentation parameters were selected based upon their ability to recapitulate the raw image and the same parameters were used on all stacks from the same case. Regions of interest (ROIs) were drawn around the EC, DG, CA1 and CA2/3 in ImageJ using Franklin & Paxinos’ mouse brain atlas (Franklin and Paxinos, 2008) as reference. The EC was further subdivided into superficial layers (layers II and III) and deep layers (layers V and VI). CA2 and CA3 were considered as one area due to the difficulty in delineating their boundaries from one another. ROIs were then cropped and saved as separate images. Counting of the number of donor cells within ROIs was automated to reduce bias and variability. To improve automated counts of donor cells, the background fluorescence of GFP images was subtracted using a 10-pixel rolling ball radius. To determine the number of donor cells within each ROI, the number of cells immuno-positive for both GFP and DAPI was calculated using a custom Python script. To ensure only GFP+ cell bodies were counted and to reduce inclusion of GFP+ neurites in the final counts, only GFP + objects colocalising with DAPI + nuclei were counted. The number of recipient cells, positive for human tau but not GFP (Tau13+/GFP−), was manually counted in all ROIs using the Cell Counter plugin in ImageJ. Colocalisation of GFP + cells with Tau13 was also performed using a custom Python script. Donor and recipient neurons were counted in at least five sections per mouse. Analysed sections were spaced by 500 μm, with each section being 50 μm thick and every 10th section used.

To account for the inherent variability associated with the AAV injection in relation to injection location, transduction efficiency and variability between mice, the number of recipient neurons was normalised to the number of transduced donor neurons. We estimated the number of GFP+/DAPI+ ‘donor’ cells required to identify one recipient cell by calculating the number of recipient cells per donor cell for each group. Mice with fewer than the threshold of donor cells to detect one recipient cell (49, the lowest group average) were excluded from the analysis as they were deemed to have too few donor cells to determine whether lack of recipient cells were due to lack of spreading or lack of available tau expressing neuron donors. The total number of mice included in the quantification of tau spread with free-floating immunohistochemistry after exclusions is shown in Table 1.

**Table 1.** Summary data of mice included in the free-floating IHC analyses.

	*ApoePOE*-KO		*APOE2*		*APOE3*		*APOE4*		Overall	
	Adult (*N* = 8)	Aged (*N* = 6)	Adult (*N* = 10)	Aged (*N* = 8)	Adult (*N* = 13)	Aged (*N* = 14)	Adult (*N* = 12)	Aged (*N* = 11)	Adult (*N* = 43)	Aged (*N* = 39)
Sex										
F	5 (62.5%)	2 (33.3%)	6 (60.0%)	4 (50.0%)	5 (38.5%)	6 (42.9%)	7 (58.3%)	8 (72.7%)	23 (53.5%)	20 (51.3%)
M	3 (37.5%)	4 (66.7%)	4 (40.0%)	4 (50.0%)	8 (61.5%)	8 (57.1%)	5 (41.7%)	3 (27.3%)	20 (46.5%)	19 (48.7%)
Age at injection (months)										
Mean (SD)	5.72 (0.258)	15.3 (0.0828)	5.64 (0.314)	15.3 (0.204)	5.49 (0.213)	15.3 (0.181)	5.53 (0.276)	15.3 (0.191)	5.58 (0.270)	15.3 (0.172)
Median [Min, Max]	5.75 [5.13, 5.93]	15.3 [15.2, 15.4]	5.64 [5.03. 6.03]	15.2 [15.2, 15.8]	5.50 [5.20, 5.80]	15.2 [15.2, 15.7]	5.60 [5.00, 5.87]	15.3 [15.2, 15.7]	5.60 [5.00, 6.03]	15.2 [15.2, 15.8]
Age at cull (months)										
Mean (SD)	8.93 (0.251)	18.5 (0.0789)	8.85 (0.325)	18.5 (10.207)	8.69 (0.190)	18.5 (0.177)	8.74 (0.247)	18.5 (0.182)	8.78 (0.260)	18.5 (0.168)
Median [Min, Max]	8.95 [8.37, 9.17]	18.5 [18.4, 16.6]	8.84 [6.20. 9.23]	18.5 [18.4, 19.0]	8.70 [8.43, 8.97]	18.5 [18.4, 18.9]	8.80 [8.30, 9.03]	18.5 [18.4, 18.9]	8.80 [8.20, 9.23]	18.5 [18.4, 19.0]
Length of incubation (months)										
Mean (SD)	3.21 (0.0185)	3.19 (0.0251)	3.21 (0.0218)	3.22 (0.0175)	3.21 (0.0289)	3.21 (0.0210)	3.21 (0.0342)	3.20 (0.0168)	3.21 (0.0267)	3.21 (0.0211)
Median [Min, Max]	3.20 [3.20, 3.24]	3 20 [3.17, 3.23]	3.20 [3.17, 3.23]	3.23 [3.20, 3.24]	3.20 [3.17, 3.27]	3.22 [3.17, 3.24]	3.20 [3.16, 3.30]	3.20 [3.17, 3.23]	3.20 [3.16, 3.30]	3.20 [3.17, 3.24]

IHC: immunohistochemistry; *APOE*: apolipoprotein E; *ApoePOE*-KO: apolipoprotein E knock-out; SD: standard deviation; F: female; M: male.

### Array tomography

Array tomography was performed using a previously established protocol (Kay et al., 2013). After perfusion, one brain hemisphere was selected at random and submerged in array tomography fixative containing 4% paraformaldehyde and 2.5% sucrose in 10 mM PBS for 2–3 h. Horizontal sections through the extent of the hippocampus were collected using a single-edged blade and forceps. The hippocampus and EC were dissected from each section and placed in array tomography fixative for a further 30 min. Samples were washed in wash buffer containing 3.5% sucrose and 50 mM glycine in 10 mM PBS for at least 5 min. Samples were dehydrated in ascending grades of cold ethanol (50%, 70%, 95%, 100%, 100% EtOH) before infiltration with ascending grades of LR White resin (Agar Scientific; 1:1 LR White:EtOH, 100% LR White, 100% LR White). Samples were kept in LR White overnight and stored at 4°C. Subsequently, individual tissue blocks were embedded in LR White within gelatin capsules (Agar Scientific, Stansted, UK; size 00) and polymerised overnight at 53°C.

Resin-embedded tissue was sectioned into ribbons of 70-nm thick serial sections using an EM UC6 Ultracut Ultramicrotome (Leica, Wetzlar, Germany), equipped with a TrimTool 45 and a Jumbo Histo Diamond Knife (DiAtome, Switzerland). Tissue ribbons were collected on glass coverslips (thickness no.1; VWR) that had been coated in a solution of 0.1% fish skin gelatin and 0.01% chromium potassium sulphate and allowed to dry overnight. For each piece of resin-embedded tissue, approximately 30–40 individual sections were collected as a ribbon. After sectioning, ribbons were allowed to dry at room temperature.

Ribbons were outlined with a hydrophobic pen, heated at 53°C for 30 min and incubated for 5 min in 50 mM glycine in Tris-buffered saline (TBS) at room temperature. To improve antibody access to epitopes, sections underwent antigen retrieval by pressure cooking for 2 min in 10 mM citric acid in ddH_2_0 (pH 6.0). After allowing ribbons to cool to room temperature, tissue was washed with TBS and incubated in glycine solution for 5 min. Sections were then blocked with array tomography block buffer, comprising 0.1% fish skin gelatin and 0.05% Tween20 in TBS, for 30 min at room temperature. Primary antibodies (anti-GFP chicken Abcam ab13970 1:200, anti-PSD-95 Rabbit Cell Signalling technologies 3450 1:100, anti-vesicular glutamate transporter 1 (VGLUT1) guinea pig AB5905 Millipore 1:200 and anti-human tau Tau13 mouse Biolegend 835201 1:100), diluted in block buffer, were applied to ribbons overnight and stored at 4°C. For each experiment, a short ribbon with known high AD pathology burden was included as a positive control to confirm the stain worked. A further short extra ribbon was used as a negative control, where application of primary antibodies was omitted, to rule out non-specific binding of secondary antibodies. For negative control ribbons, primary antibody solution was replaced by array tomography block buffer. The following day, ribbons were thoroughly washed in TBS and secondary antibodies (goat anti-chicken AlexaFluor 488 Abcam ab150169, donkey anti-rabbit AlexaFluor 594 Invitrogen A-21207, goat anti-mouse AlexaFluor 647 Invitrogen A-21240 and goat anti-guinea pig AlexaFluor 405), diluted 1:50 in array tomography block, were applied for 30 min at room temperature. Secondary antibodies were removed by washing thoroughly with TBS and sections were counterstained with 0.01 mg/m DAPI (Sigma-Aldrich, St. Louis, MO, USA) for 5 min. Array ribbons were again washed in TBS and finally mounted onto glass slides (VWR) with Immu-mount™ mounting media (Thermo Fisher Scientific). To limit introduction of bias, array ribbons were stained in batches whereby each batch contained an equal proportion of each mouse genotype.

Images were acquired at the same position on each section of the ribbon using a ZEISS Axio Imager Z2 epifluorescent microscope equipped with a CoolSnap digital camera and array tomography macros (Carl Zeiss). Initially, a tile scan of the entire ribbon was obtained at 10× magnification. ROIs were identified and nuclei in two adjacent sections were selected, resulting in the generation of a position list containing co-ordinates for the corresponding location in each serial section. Images were acquired in the middle molecular layer (MML) of the DG where synaptic terminals from EC layer II cells are located. Images were also taken in the synaptic input layers of CA1 or CA2/3. When imaging each batch of ribbons, exposure times for each channel were maintained to enable unbiased comparisons. Mice in which GFP staining could not be observed were excluded from the study. Group sizes for the array tomography study are shown in Table 2.

**Table 2.** Summary data of mice included in the array tomography analyses.

	*ApoePOE*-KO		*APOE2*		*APOE3*		*APOE4*		Overall	
	Adult (*N* = 6)	Aged (*N* = 6)	Adult (*N* = 6)	Aged (*N* = 3)	Adult (*N* = 6)	Aged (*N* = 7)	Adult (*N* = 4)	Aged (*N* = 6)	Adult (*N* = 22)	Aged (*N* = 22)
Sex										
F	2 (33.3%)	4 (66.7%)	2 (33.3%)	0 (0%)	3 (50.0%)	3 (42.9%)	2 (50.0%)	3 (50.0%)	9 (40.9%)	10 (45.5%)
M	4 (66.7%)	2 (33.3%)	4 (66.7%)	3 (100%)	3 (50.0%)	4 (57.1%)	2 (50.0%)	3 (50.0%)	13 (59.1%)	12 (54.5%)
Age at injection										
Mean (SD)	5.65 (0.268)	15.4 (0.147)	5.48 (0.0757)	15.2 (0.0513)	5.33 (0.197)	15.4 (0.213)	5.53 (0.370)	15.4 (0.231)	5.49 (0.248)	15.4 (0.187)
Median [Min, Max]	5.69 [5.13, 5.90]	15.4 [15.3, 15.7]	5.47 [5.40, 5.60]	15.2 [15.2, 15.3]	5.27 [5.20, 5.73]	15.3 [15.2, 15.7]	5.62 [5.00, 5.87]	15.4 [15.2, 15.7]	5.52 [5.00, 5.90]	15.3 [15.2, 15.7]
Age at cull										
Mean (SD)	8.85 (0.252)	18.6 (0.157)	8.61 (0.210)	18.5 (0.0404)	8.56 (0.172)	18.6 (0.217)	8.74 (0.311)	18.6 (0.223)	8.69 (0.248)	18.6 (0.186)
Median [Min, Max]	8.89 [8.37, 9.10]	18.6 [18.5, 18.9]	8.67 [8.20, 8.80]	18.4 [18.4, 18.5]	8.50 [8.43, 8.90]	18.5 [18.4, 18.9]	8.82 [8.30, 9.03]	18.6 [18.4, 18.9]	8.69 [8.20, 9.10]	18.5 [18.4, 18.9]
Incubation										
Mean (SD)	3.21 (0.0163)	3.19 (0.0164)	3.13 (0.226)	3.21 (0.0173)	3.22 (0.0319)	3.20 (0.0207)	3.22 (0.0597)	3.21 (0.0226)	3.19 (0.120)	3.20 (0.0207)
Median [Min, Max]	3.20 [3.20, 3.24]	3.19 [3.17, 3.20]	3.22 [2.67, 3.24]	3.20 [3.20, 3.23]	3.23 [3.17, 3.26]	3.20 [3.17, 3.23]	3.20 [3.16, 3.30]	3.20 [3.17, 3.23]	3.20 [2.67, 3.30]	3.20 [3.17, 3.23]
Stack length										
Mean (SD)	21.7 (4.50)	24.2 (6.68)	25.0 (5.18)	23.7 (1.53)	23.5 (6.60)	23.3 (5.31)	23.5 (4.36)	25.5 (4.51)	23.4 (5.09)	24.2 (4.95)
Median [Min, Max]	22.5 [15.0, 26.0]	27.5 [13.0, 30.0]	26.0 [18.0, 30.0]	24.0 [22.0, 25.0]	23.0 [13.0, 31.0]	23.0 [16.0, 32.0]	22.5 [20.0, 29.0]	26.5 [18.0, 31.0]	24.0 [13.0, 31.0]	25.0 [13.0, 32.0]

*APOE*: apolipoprotein E; *ApoePOE*-KO: apolipoprotein E knock-out; SD: standard deviation; F: female; M: male.

Serial sequences of images were converted into stacks using ImageJ and aligned with a rigid and affine registration via an in-house MATLAB script. Aligned stacks were edited such that out of focus slices at either end were removed, out of focus areas were cropped out, rotation of the stack was corrected, and any debris was filtered from the image. Using a custom MATLAB script, individual channels of the stack were segmented using a semi-automatic local threshold, based on mean values. Using these scripts, only objects appearing in the same location in two or more sequential slices (i.e. 3D objects) were included in the segmented stack, thus helping to reduce non-specific signals. Segmentation parameters were selected based upon their ability to recapitulate the raw image, and the same parameters were used on all stacks from the same staining batch to reduce bias. Segmented images were then run through custom Python scripts to calculate the density of objects per mm^3^ of tissue. Here, the number of objects in the neuropil was quantified after removing confounding structures, such as blood vessels. Following this, colocalisation between markers of interest was determined using custom Python scripts. Here, objects were considered colocalised when ⩾25% of an objects volume was occupied by the colocalising object. For pre- and post-synaptic objects to be considered a synaptic pair, the distance between their centroids had to be <0.5 μm. Python scripts can be found at https://github.com/lewiswilkins/Array-Tomography-Tool.

### Statistical analyses

Statistical analyses were performed using R version 4.1.2 in RStudio (The R Foundation, n.d.). To estimate sample size, we used the wp.kanova package with effect size data calculated from Dujardin et al. (2022) who used the same virus injected in mice of different background strains and examined similar tau spread to uninfected neurons. This power calculation estimated a required *n* of 4 per group. We injected initially at least 10 per genotype at each age to allow for examining sex effects and to account for potentially subtler effects between APOE genotypes on the same background strain. Not all mice that received AAV injection could be included in the study. Mice were excluded from the study if there was no GFP signal in EC or with less than 49 GFP-positive donor cells in EC (the minimum calculated in this study to be required to identify one recipient cell). Mice were excluded from the array tomography study if no tissue blocks embedded for array tomography contained GFP-expressing presynapses.

Multiple linear regression (‘stats’ package) was used to analyse differences in the total estimated number of donors and recipients throughout the entire EC and hippocampus (non-normalised and normalised), the percentage of donor cells colocalising with human tau, and the total estimated number of recipient cells generated through local and circuit-based spread (non-normalised and normalised). Analyses of variance (ANOVAs) (‘stats’ package) were performed on multiple linear regression models to determine main effects (*APOE* genotype, age and sex) and interactions between main effects (*APOE* genotype × age).

Linear mixed-effect models (‘lmerTest’ package) were used to analyse the effect of ROI, or brain region, on the total estimated number of donor and recipient cells. Linear mixed-effect models were selected as they can account for the non-independent, hierarchical structure of the data (i.e. multiple measurements obtained within different brain regions from the same animal) by allowing for both fixed and random effects. Use of this method increases statistical power as the need to aggregate data is eliminated. ANOVAs (‘stats’ package) were performed on mixed-effect models to determine the main effects of each fixed effect and interactions between main effects. Fixed effects included *APOE* genotype, age group, brain region and sex.

Multiple linear regression and linear mixed-effect models assume linearity, normal distribution of residuals and homogeneity of variance. Linearity was assessed by plotting model residuals against predictors, distribution of residuals was checked with a QQ-plot, and homogeneity of variance checked by plotting residuals against fitted values. If the model did not meet assumptions, data were transformed using the Tukey or Box–Cox transformations. Post hoc testing was conducted for simple pairwise comparisons and utilised estimated marginal means (‘emmeans’ package) with the Tukey correction for multiple testing. Even though some of our models did not perfectly meet all assumptions, recent work suggests these statistical models are robust to violation of assumptions (Schielzeth et al., 2020).

Results were considered statistically significant at *p* < 0.05. Full data spreadsheets, statistical outputs and models used in all analyses are provided in Supplementary data (Figure 3, standard immunohistochemistry experiments data in Supplemental data 1, R Notebook statistical analysis script in Supplemental data 2; Figure 5 data in Supplemental data 3, R Notebook statistical analysis script in Supplemental data 4).

## Results

**Figure 2.**
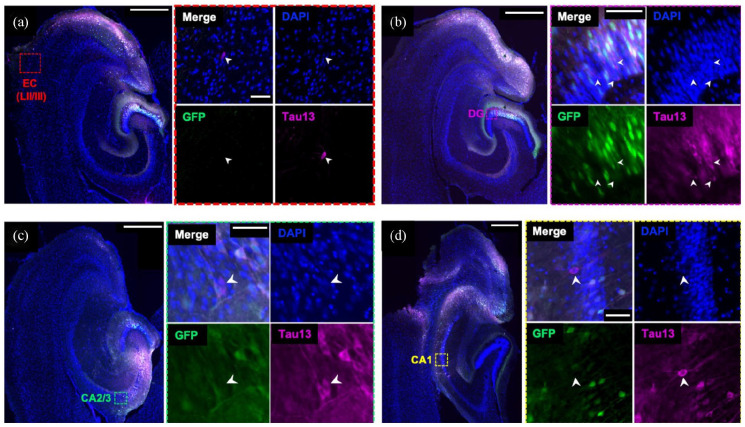
Tau spreads to cell bodies of non-infected cells in mouse brain. AAV was injected into entorhinal cortex (EC). Recipient cells (GFP−/Tau13+), shown with white arrowheads, were observed in the EC (a), indicative of local propagation of tau. Recipient cells were also observed in downstream brain regions, including in DG (b), CA2/3 (c) and CA1 (d), suggestive of spread through anatomically connected circuits. Boxes in (a) to (d) show locations of magnified regions in insets. Sections were stained for DAPI (blue), GFP (green) and Tau13 (magenta). Scale bars represent 500 mm in large panels and 50 mm in insets.

Human mutant tau (P301L) was injected into the EC of *APOE* TR and *Apoe* KO mice using AAV gene delivery. This AAV-mediated method was previously used to investigate tau spread (Wegmann et al., 2015, 2019) and allows for discrimination of ‘donor cells’, transduced with the viral construct, and ‘recipient cells’, to which human tau has spread, through simple immunohistochemical techniques (Figure 2). Here, we injected adult (5–6 months of age) and aged (15–16 months of age) mice to investigate whether *APOE* isoform or ageing influenced tau spread. We observed GFP-positive and tau-positive ‘donor cells’ predominantly in EC, but donor cells were also observed in downstream hippocampal regions DG and CA1 and CA2/3 (Figure 2).

**Figure 3.**
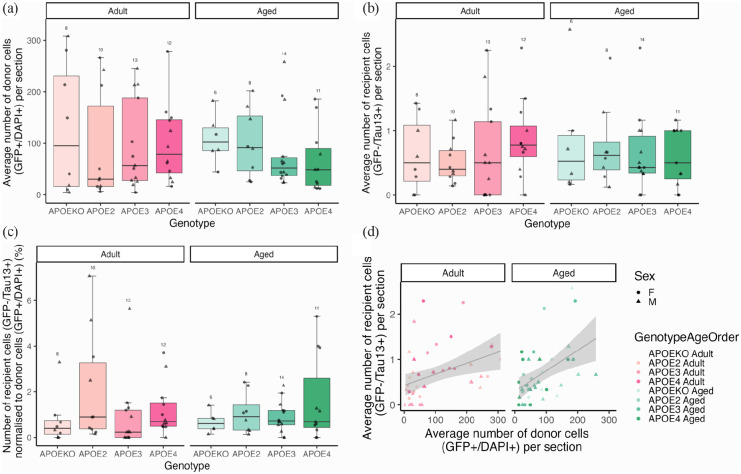
Quantification tau spread to non-infected cell bodies. For each mouse, the average number of GFP-positive AAV expressing cells per section (a), human tau-positive, GFP-negative recipient cells per section (b) and the percentage of recipient cells normalised to donor cells (c) were calculated. No differences between APOE genotype, age or sex were found. A positive correlation between the number of donor cells and recipient cells (d) was observed. Each data point represents the average value per mouse with the number of mice per group noted above the boxes in (a) to (c).

Due to the inherent variability associated with stereotaxic AAV injection, in relation to injection location, transduction efficiency and mouse-to-mouse variability, it was important to first establish whether there were differences in the total estimated number of donor cells between groups. Overall, genotype (*F*[3,73] = 0.12, *p* = 0.94), age (*F*[1,73] = 0.42, *p* = 0.52) and sex (*F*[1,73] = 0.03, *p* = 0.86) did not impact the average number of donor cells per section throughout the EC and hippocampal formation (Figure 3(a), ANOVA after linear model ~ Genotype * Age + Sex, Box–Cox transformed data). There was also no interaction between genotype and age (*F*[3,73] = 1.63, *p* = 0.19), indicating that there is insufficient evidence to reject the null hypotheses that the different genotypes, ages and sexes do not differ in tau virus expression.

While human tau accumulation in neuronal cell bodies not expressing AAV was observed in 70 out of 82 animals in the study (85%), we observed very few recipient cells per section – on average less than one tau positive, GFP cell per section with 5–15 sections analysed per mouse (Figure 3(a)). This small amount of tau spread to GFP-negative neuronal cell bodies did not differ significantly by genotype (*F*[3,73] = 0.28, *p* = 0.84), age (*F*[1,73] = 0.07, *p* = 0.79) or sex (*F*[1,73] = 0.93, *p* = 0.34) (Figure 3(b), ANOVA after linear model ~ Genotype * Age + Sex, Tukey transformed data). When the average number of recipient cells was normalised to the average number of donor cells per section for each animal, we observed a similar pattern of no effect of APOE genotype (*F*[3,73] = 1.97, *p* = 0.13), age (*F*[1,73] = 0.23, *p* = 0.63) or sex (*F*[1,73] = 0.93, *p* = 0.43) on tau spread to neuronal cell bodies not expressing GFP (Figure 3(c), ANOVA after linear model ~ Genotype * Age + Sex, Tukey transformed data). As expected, the number of recipient cells was positively correlated with the number of donor cells (Figure 3(d), correlation *S* = 43,610, rho = 0.53, *p* < 0.0001).

**Figure 4.**
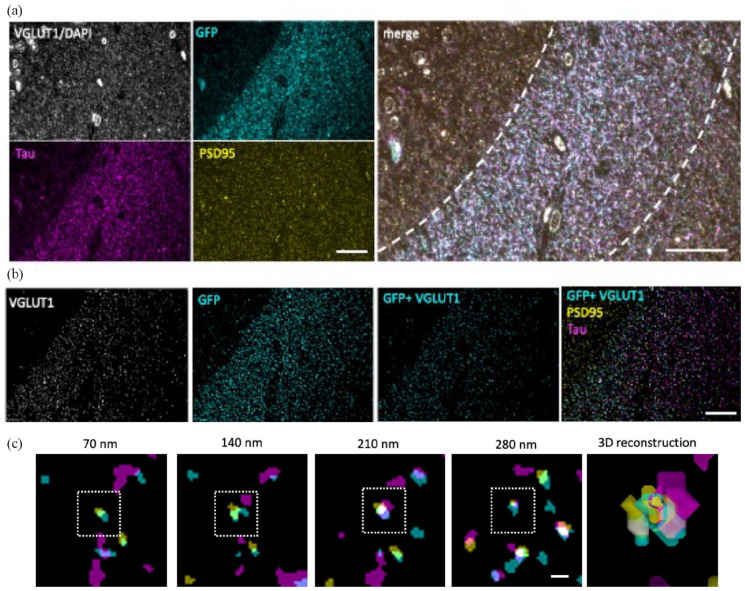
Array tomography imaging. Representative raw images from a single section of an array tomography ribbon (a) show staining for VGLUT1 and DAPI (grey), GFP (cyan), human tau (magenta) and PSD95 (yellow). Raw image stacks were aligned and segmented to exclude any objects present in single sections and to exclude DAPI staining by implementing a size filter. To find pre-synaptic terminals positive for GFP, segmented VGLUT1 and GFP images were multiplied then compared with PSD95 and tau channels (b). Tau-positive post-synapses apposed to GFP-positive presynapses were observed in three-dimensional images. Images of sequential 70 nm sections are shown in (c) with a box indicating a post-synaptic density containing tau. Scale bars represent 20 µm in (a) and (b) and 1 µm in (c).

To determine whether human tau spreads from pre- to post-synaptic terminals in our mouse model, we used high-resolution array tomography imaging (Figure 4). Images from sequential 70 nm serial sections were aligned and segmented, excluding objects present in only a single section which are likely to be noise. The segmented VGLUT1 labelled pre-synaptic terminals that colocalised with GFP staining were considered potential ‘donor’ presynapses as GFP-expressing neurons also express human tau. Post-synaptic densities were labelled with PSD95, and segmented PSD puncta within 0.5 mm of a GFP-positive pre-synapse were considered post-synaptic partners and potential ‘recipient’ post-synapses. We observed trans-synaptic tau spread of human tau protein from GFP-containing pre-synaptic terminals (arising from neurons expressing human tau) to apposed post-synaptic densities in all groups examined (Figure 4).

**Figure 5.**
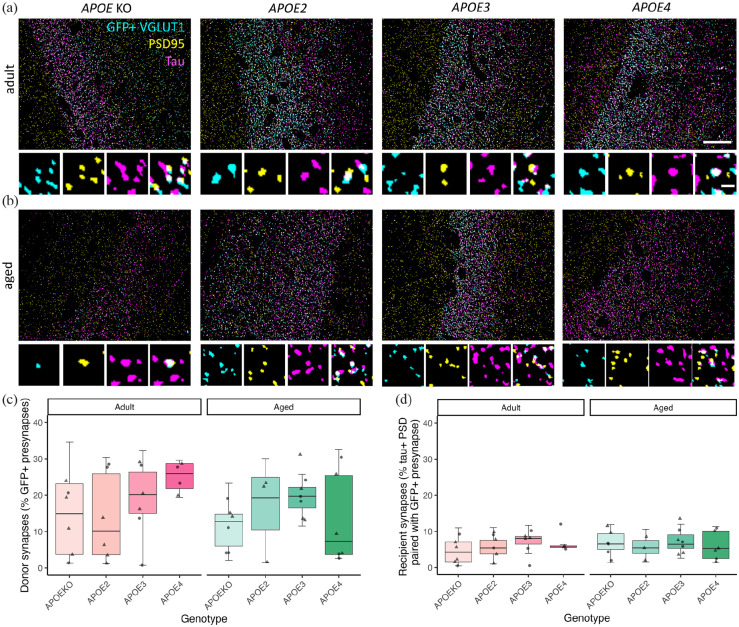
Trans-synaptic tau spread observed in all groups. Representative segmented images from a single section of array tomography ribbons from adult (a) and aged (b) animals show GFP-positive VGLUT1 presynapses (cyan), human tau (magenta) and PSD95 (yellow). Large panels show an overview of all channels with zoomed in separate channels and merge below illustrating synaptic tau. Analyses revealed no difference in the percentage of presynapses containing GFP (c) or in recipient synapses where tau has spread to the post-synapse (d) between *APOE* genotypes or ages. Scale bars represent 20 µm in large panels and 1 µm in small panels.

On average, 16% of synaptic pairs (defined as a VGLUT1-positive puncta within 0.5 mm of a PSD95 positive puncta) had GFP colocalised with VGLUT1, meaning they are presynapses arising from neurons expressing human tau and GFP AAV. This proportion of pre-synaptic terminals expressing tau did not differ by genotype (*F*[3,34.78] = 1.76, *p* = 0.33), age (*F*[1,34.78] = 0.53, *p* = 0.47) or sex (*F*[1,34.8] = 1.23, *p* = 0.31, ANOVA after linear mixed-effects model on Box–Cox transformed data ~ Genotype * Age + Sex+ (1|animal); Figure 5); however, it should be noted that one of our genotypes in the aged group (*APOE2* genotype) did not have any female mice with blocks containing DG samples suitable for AT analyses and several other groups had only two mice of each sex, thus our lack of sex effects should be interpreted with caution. 3.5% of post-synaptic densities that are within 0.5 mm of a donor synapse contained tau. This readout of pre- to post-synaptic tau spread did not differ by genotype (*F*[3,34.11] = 0.59, *p* = 0.62), age (*F*[1,34.11] = 0.07, *p* = 0.80) or sex (*F*[1,34.16] = 0.64, *p* = 0.43, ANOVA after linear mixed-effects model on Box–Cox transformed data ~ Genotype * Age + Sex + (1|animal); Figure 5).

## Discussion

Pathological tau propagates through neuroanatomically connected circuits in AD and mouse models (Clavaguera et al., 2013; De Calignon et al., 2012; DeVos et al., 2018; Liu et al., 2012; Vogel et al., 2020; Wegmann et al., 2015), potentially via synapses (Calafate et al., 2015; Dujardin et al., 2014; Pickett et al., 2017). Progressive accumulation and spread of tau pathology in the AD brain are intimately associated with neurodegeneration and cognitive symptoms (Bejanin et al., 2017; Giannakopoulos et al., 2003), highlighting tau propagation as a potential therapeutic target. In animal models of tau spread, human tau propagated from EC neurons to DG granule cells, via the perforant path, and to other synaptically connected hippocampal regions (Asai et al., 2015; De Calignon et al., 2012; Pickett et al., 2017). Local propagation to neighbouring neurons within the EC was also observed (De Calignon et al., 2012; Wegmann et al., 2019). As expected, in our AAV-mediated model of tau spread, recipient cells were observed in hippocampal regions, suggestive of propagation across synaptically connected EC–hippocampal circuitry. Recipient cells were also seen within the EC, adjacent to donor cells expressing GFP and mutant human tau, indicating local spread of human tau. Although not investigated, local propagation could also be mediated by synaptic mechanisms, due to the many recurrent collaterals within the EC (Canto et al., 2008).

Indirect evidence has indicated *APOE* genotype might specifically affect tau spread (Corder et al., 2004; Ghebremedhin et al., 1998; Shi et al., 2017). In our model of tau spread, *APOE* genotype had no effect on propagation of tau throughout the whole EC and hippocampus. Local and circuit-based tau spread may employ different and altered balance of propagation mechanisms (Brunello et al., 2020), which in turn might be variably influenced by APOE isoforms. In addition, tau propagation was not impacted upon *APOE*-KO, indicating *APOE* was not required for spread of tau through the living brain. As a secondary outcome measure, we did not see any effect of mouse sex on tau propagation, in line with previous studies (Dujardin et al., 2022); however, our group sizes were inadequate when split by sex to draw firm conclusions.

Our findings align with previous work in mice, where AAV-induced *APOE4* expression had no effect on tau pathology (Koller et al., 2020), and some studies in humans that found no *APOE* genotype effects on extent of tau pathology (Lautner et al., 2014; Morris et al., 2010). However, this work contrasts findings from animal models where both *APOE4* and *APOE2* exacerbated tau pathology (Andrews-Zwilling et al., 2010; Shi et al., 2017; Zhao et al., 2018) and work in humans finding *APOE4* to exacerbate progression of tau pathology (Baek et al., 2020; Sabbagh et al., 2013; Therriault et al., 2021). While differences in animal models likely contribute to discrepancies, it is also possible that *APOE*-related exacerbation of tau pathology in mouse models is not a direct result of enhanced tau spread. Interestingly, some studies finding associations between tau pathology and *APOE* genotype found effects to be dependent on or exacerbated by the presence of Aβ pathology (Farfel et al., 2016; Hohman et al., 2018; Ramanan et al., 2019; Salvadó et al., 2022). While *APOE4* has been found to aggravate tau hyperphosphorylation and tau-mediated neurodegeneration independent of Aβ in model systems (Brecht et al., 2004; Lin et al., 2018; Litvinchuk et al., 2021; Shi et al., 2017; Tesseur et al., 2000; Wang et al., 2018), the presence of Aβ accelerates tau propagation from the EC to synaptically connected regions (Pooler et al., 2015). Consequently, *APOE* effects on tau propagation may be minimal in the absence of Aβ, as in our model, although this remains to be investigated.

The current AAV-mediated approach was similar to that used in previous study of tau propagation (Pickett et al., 2017; Wegmann et al., 2015, 2019). However, we observed far fewer recipient cells. In WT mice, Wegmann et al. (2019) identified an average 2.5–12 recipient neurons per brain section per mouse. In contrast, we observed an average of less than one recipient cell per brain section per mouse. It is possible that observation of so few recipient cells limited our ability to detect subtle differences between *APOE* genotypes. In stark contrast to our findings, Wegmann et al. (2019) found tau spread to be accelerated in aged mice. Our finding of no difference in tau propagation between adult and aged mice was surprising when considering AD is a disease of ageing, that progression of tau pathology is associated with increasing age (Braak et al., 2011) and that age dependence of tau propagation has also been reported in *Drosophila* (Aqsa and Sarkar, 2021). Various factors may have contributed to this discrepancy, like age differences between the mice in the respective studies, methodological differences in cell counting and differences in AAV serotypes.

Tau spread may also be facilitated by non-cell autonomous mechanisms, for example, by glial cells. Microglia and astrocytes have been implicated in tau propagation in some tau spread models (Asai et al., 2015; De Calignon et al., 2012), but not others (Wegmann et al., 2015). APOE is mainly synthesised by glial cells in the human brain (Kang et al., 2018; Zhang et al., 2014) and it modulates glial responses in AD (Tzioras et al., 2019). Understanding whether glia take up tau in our model and the role of *APOE* in this will be important. Microglia are capable of releasing tau-containing vesicles both in vivo (Clayton et al., 2021) and in vitro (Brelstaff et al., 2021; Hopp et al., 2018), suggesting a potential role in tau spread. In addition, the APOE receptor low-density lipoprotein receptor–related protein 1 (LRP1), which is expressed both by neurons and glia (Liu et al., 2017; Yang et al., 2016), acts as a master regulator of tau spread (Rauch et al., 2020). Understanding whether *APOE* genotype modulates this protein in the context of tau propagation may shed further light on mechanistic underpinnings of tau spread and AD risk.

## Conclusion

In conclusion, in our model, we observed successful transfer of tau via synaptic systems, but *APOE* genotypes did not play a significant role in tau transduction from the EC to CA1, CA2/3 and DG.

## Supplemental Material

sj-csv-1-bna-10.1177_23982128231191046 – Supplemental material for Apolipoprotein E isoform does not influence trans-synaptic spread of tau pathology in a mouse model Click here for additional data file. Supplemental material, sj-csv-1-bna-10.1177_23982128231191046 for Apolipoprotein E isoform does not influence trans-synaptic spread of tau pathology in a mouse model by Caitlin Davies, Jane Tulloch, Ellie Yip, Lydia Currie, Marti Colom-Cadena, Susanne Wegmann, Bradley T Hyman, Lewis Wilkins, Monique Hooley, Makis Tzioras and Tara L Spires-Jones in Brain and Neuroscience Advances

sj-csv-3-bna-10.1177_23982128231191046 – Supplemental material for Apolipoprotein E isoform does not influence trans-synaptic spread of tau pathology in a mouse model Click here for additional data file. Supplemental material, sj-csv-3-bna-10.1177_23982128231191046 for Apolipoprotein E isoform does not influence trans-synaptic spread of tau pathology in a mouse model by Caitlin Davies, Jane Tulloch, Ellie Yip, Lydia Currie, Marti Colom-Cadena, Susanne Wegmann, Bradley T Hyman, Lewis Wilkins, Monique Hooley, Makis Tzioras and Tara L Spires-Jones in Brain and Neuroscience Advances

sj-pdf-5-bna-10.1177_23982128231191046 – Supplemental material for Apolipoprotein E isoform does not influence trans-synaptic spread of tau pathology in a mouse model Click here for additional data file. Supplemental material, sj-pdf-5-bna-10.1177_23982128231191046 for Apolipoprotein E isoform does not influence trans-synaptic spread of tau pathology in a mouse model by Caitlin Davies, Jane Tulloch, Ellie Yip, Lydia Currie, Marti Colom-Cadena, Susanne Wegmann, Bradley T Hyman, Lewis Wilkins, Monique Hooley, Makis Tzioras and Tara L Spires-Jones in Brain and Neuroscience Advances

sj-Rmd-2-bna-10.1177_23982128231191046 – Supplemental material for Apolipoprotein E isoform does not influence trans-synaptic spread of tau pathology in a mouse model Click here for additional data file. Supplemental material, sj-Rmd-2-bna-10.1177_23982128231191046 for Apolipoprotein E isoform does not influence trans-synaptic spread of tau pathology in a mouse model by Caitlin Davies, Jane Tulloch, Ellie Yip, Lydia Currie, Marti Colom-Cadena, Susanne Wegmann, Bradley T Hyman, Lewis Wilkins, Monique Hooley, Makis Tzioras and Tara L Spires-Jones in Brain and Neuroscience Advances

sj-Rmd-4-bna-10.1177_23982128231191046 – Supplemental material for Apolipoprotein E isoform does not influence trans-synaptic spread of tau pathology in a mouse model Click here for additional data file. Supplemental material, sj-Rmd-4-bna-10.1177_23982128231191046 for Apolipoprotein E isoform does not influence trans-synaptic spread of tau pathology in a mouse model by Caitlin Davies, Jane Tulloch, Ellie Yip, Lydia Currie, Marti Colom-Cadena, Susanne Wegmann, Bradley T Hyman, Lewis Wilkins, Monique Hooley, Makis Tzioras and Tara L Spires-Jones in Brain and Neuroscience Advances
