# Supplementary material for: Apolipoprotein E isoform does not influence trans-synaptic spread of tau pathology in a mouse model
Source: Brain Neurosci Adv. 2023 Aug 17;7:23982128231191046. doi: 10.1177/23982128231191046 (PMC10433884; doi:10.1177/23982128231191046)
Supplement: sj-pdf-5-bna-10.1177_23982128231191046 – Supplemental material for Apolipoprotein E isoform does not influence trans-synaptic spread of tau pathology in a mouse model [file sj-pdf-5-bna-10.1177_23982128231191046.pdf]

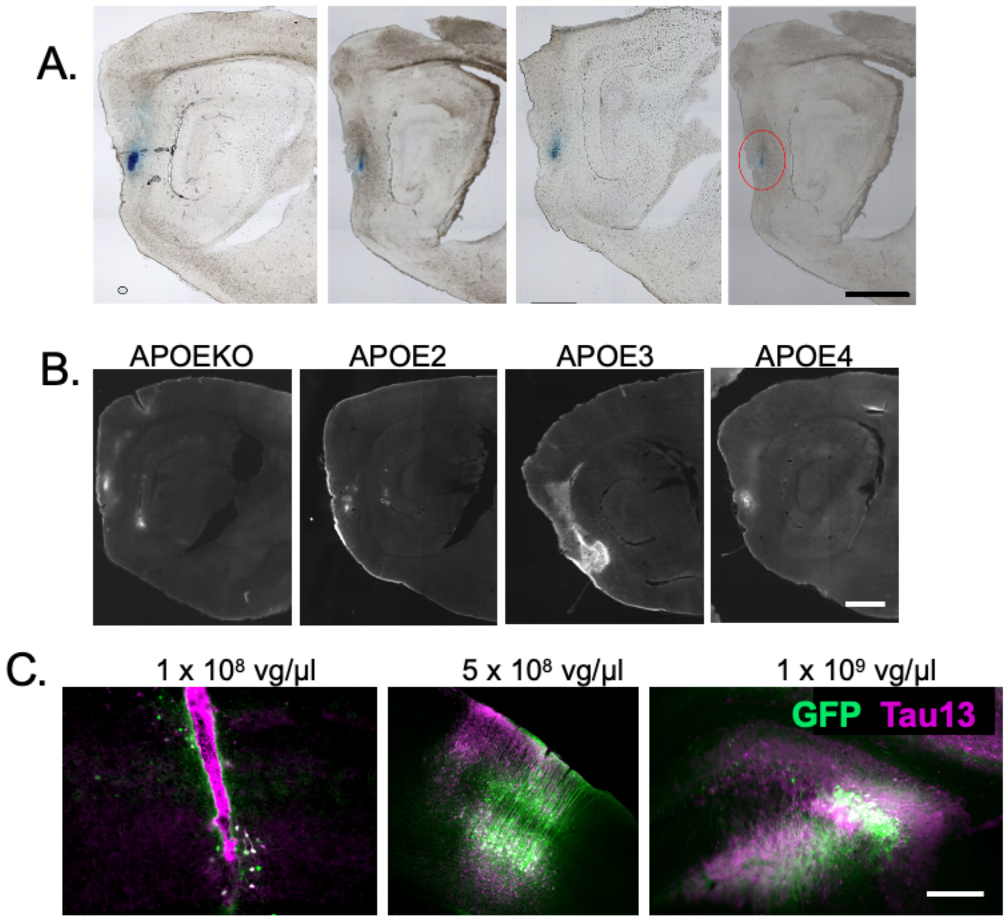

Supplementary figure 1: Pilot injections. To determine accurate stereotaxic coordinates that consistently targeted superficial layers of the EC, a blue (**A**) and fluorescent dye (**B**) were injected into the EC using a range of co-ordinates. In multiple WT mice (**A** and **B**), co-ordinates: A/P = -4.7mm, M/L = ±4.25mm, D/V = -2.50mm; with micropipette tilted 20°, consistently and accurately targeted EC LII/III (**A**; blue spots of dye). Comparison to brain atlas confirmed accuracy in the EC (**B**; red circle & green bar). To ensure any differences between WT and *APOE*-TR mice did not influence injection accuracy, co-ordinates were further confirmed in *APOE*-TR and APOE KO mice (**B**). C. Three different concentrations ($1x10^8$ vg/µl , $5x10^8$ vg/µl , and$1x10^9$ vg/µl) of GFP-2a-Tau(P301L) AAV were bilaterally injected into the somatosensory cortices of three C57/BL6 mice. Mice were culled after 3 weeks, to allow time for viral expression, and brains were sectioned in the coronal plane. Sections were then stained for GFP and human tau (Tau13). AAV expression was confirmed by presence of GFP+/Tau13+ cells at all three tested concentrations. Injection with the viral concentration of $1x10^8$ vg/µl resulted in minimal transduction of cells around the injection site. Concentrations of $5x10^8$ vg/µl and $1x10^9$ vg/µl resulted in a greater number of transduced cells. The concentration of $5x10^8$ vg/µl was selected for use. Scale bars represent 500 µm in A and B, 200 µm in C, vg = viral genomes.
